# Clinical Outcomes of Balloon Kyphoplasty for Osteoporotic Vertebral Fractures With Posterior Element Injury: A Retrospective Study

**DOI:** 10.7759/cureus.113144

**Published:** 2026-07-22

**Authors:** Akihisa Takami, Keigo Shirasaki, Kenya Isizu, Tetsuya Watanabe, Keiichiro Iida

**Affiliations:** 1 Orthopedic Surgery, Shimonoseki City Hospital, Shimonoseki, JPN; 2 Orthopedic Surgery, Shimonoseki City Hospital, Fukuoka, JPN

**Keywords:** adjacent vertebral fracture (avf), balloon kyphoplasty (bkp), osteoporotic vertebral fracture (ovf), outcome, posterior element injury

## Abstract

Introduction

Balloon kyphoplasty (BKP) has been shown to be effective in treating osteoporotic vertebral fractures (OVFs); however, its applicability in cases with posterior element injury remains unclear. Posterior element injuries may reflect underlying spinal instability, which could affect clinical outcomes. This study aimed to evaluate the efficacy of BKP in OVFs complicated by posterior element injury.

Materials and methods

We retrospectively analyzed 18 of 189 patients who underwent BKP between 2012 and 2023, in whom posterior element injury was confirmed by magnetic resonance imaging (MRI). Pain improvement at three months postoperatively was assessed using the numerical rating scale (NRS), and the presence of adjacent vertebral fractures (AVFs) was recorded. Patients with both NRS < 4 and no AVFs were classified as having a favorable outcome. Clinical and radiographic factors, including age, sex, fracture location, bone mineral density, fracture history, kyphotic angle, and vertebral instability, were compared between favorable and unfavorable outcome groups.

Results

The mean age of the 18 patients was 82 ± 6 years; there were six males and 12 females. At three months after BKP, eight patients (44%) had NRS ≥ 4, and seven patients (39%) developed AVFs. Vertebral instability ≥10° was significantly more common in the unfavorable outcome group than in the favorable outcome group (10/11 (91%) vs. 2/7 (29%), p = 0.006).

Conclusions

BKP can still be effective for OVFs with posterior element injury; however, its efficacy appears limited in cases with significant vertebral instability. Alternative stabilization strategies may be considered in such cases.

## Introduction

Osteoporotic vertebral fractures (OVFs) are a common and serious condition in the elderly, often resulting in severe back pain, decreased mobility, and reduced quality of life. Balloon kyphoplasty (BKP) is a widely used minimally invasive treatment that has been shown to provide rapid pain relief and functional improvement in selected patients [1-3].

Traditionally, BKP is indicated for OVFs involving collapse of the anterior column without disruption of the posterior vertebral wall or neural compromise. However, recent reports suggest that even cases with limited middle column injury may respond favorably to BKP, provided neurological symptoms are absent [4, 5]. Despite these expanded indications, the role of BKP in patients with posterior element injury remains unclear. Injuries to the posterior elements-such as spinous process fractures-are often subtle and easily missed, particularly when not actively sought on magnetic resonance imaging (MRI) in cases of OVFs. These injuries may represent occult spinal instability [6], raising concerns about the appropriateness of BKP in such cases. Clarifying whether BKP is appropriate for OVFs with posterior element injury could contribute to more accurate surgical decision-making and help avoid inappropriate procedures.

This study aimed to assess the efficacy of BKP in patients with OVFs complicated by posterior element injury and to explore whether these injuries may influence clinical outcomes or necessitate alternative surgical strategies.

## Materials and methods

This retrospective cohort study was conducted at Shimonoseki City Hospital, Japan. Consecutive patients who underwent BKP for OVFs between January 2012 and December 2023 were screened for eligibility. The study protocol was approved by the Ethics Committee of Shimonoseki City Hospital (Approval No. 2024SCHEC-37). Informed consent was obtained through an opt-out process in accordance with institutional guidelines.

**Figure 1:**
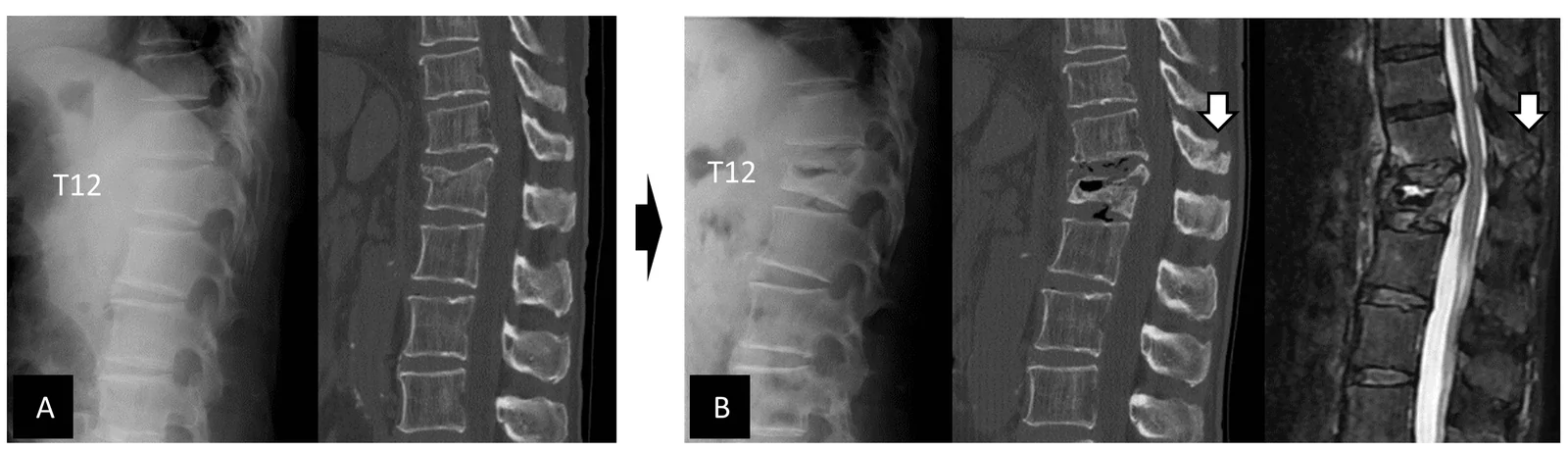
Serial images demonstrating delayed development of a spinous process fracture during progression of vertebral collapse A) Radiograph and computed tomography (CT) at two days post-injury. A fracture of the T12 vertebral body is observed with no injury to the posterior structures; B) Radiograph, CT, and magnetic resonance imaging (MRI) at five months post-injury. A cleft formation is noted in the T12 vertebral body. A vertical fracture is observed in the spinous process of T11 (white arrow).

Among 230 patients who underwent BKP during the study period, patients with neurological deficits or less than three months of follow-up were excluded. Of the remaining 189 patients, 18 with posterior element injury confirmed on preoperative MRI met the inclusion criteria and were included in this study (Figure 1).

All posterior element injuries in this study were spinous process fractures. The spinous process fractures were diagnosed using preoperative T1-weighted, T2-weighted, and STIR MRI sequences. The MRI images were reviewed by two orthopedic surgeons. We assessed the baseline characteristics (age, sex, fracture level, bone mineral density (BMD), and previous vertebral fractures) and radiographic parameters (kyphotic angle, vertebral instability, fracture line visibility on computed tomography (CT), and location of spinous process fracture) of these 18 patients. BMD was measured using dual-energy X-ray absorptiometry (DEXA) at the hip. Kyphotic angle was measured in the weight-bearing position, and vertebral instability was defined as the difference in kyphotic angle between sitting and supine positions [7]. To better characterize patients with posterior element injury, baseline demographic and radiographic characteristics were also compared with those of the remaining 171 patients who underwent BKP without posterior element injury. The primary outcome was a favorable clinical outcome at three months after BKP, defined as both a numerical rating scale (NRS) pain score <4 and the absence of adjacent vertebral fractures (AVFs) [8]. Because AVF is one of the most common complications after BKP and may adversely affect subsequent clinical outcomes, we used this composite outcome to provide a more comprehensive assessment of treatment success. An exploratory analysis was performed to compare potential prognostic factors between the favorable and unfavorable outcome groups. Clinical outcomes were assessed at three months after BKP, and complete follow-up data were available for all 18 patients. Final follow-up outcomes were not evaluated in this study.

Because this was an exploratory retrospective study, no formal sample size calculation was performed. All eligible patients meeting the inclusion criteria during the study period were included in the analysis. Continuous variables were categorized using cut-off values based on previous studies [9]. Categorical variables were analyzed using Fisher's exact test. A p-value <0.05 was considered statistically significant. Statistical analyses were performed using SPSS version 29.0 (IBM Inc., Armonk, NY, USA).

## Results

A total of 18 patients with OVFs and posterior element injury confirmed on MRI were included in this study. The mean age was 82 ± 6 years, and 12 patients (67%) were female. Most fractures were located at the thoracolumbar junction, particularly at T12 and L1. The mean BMD was 0.64 ± 0.13 g/cm². The mean local kyphotic angle was 24 ± 9°, and the mean vertebral instability was 13 ± 6°. Spinous process fractures were located one level above the fractured vertebra in 15 patients (83%) and at the same level in three patients (17%). CT demonstrated a fracture line in the spinous process in 11 patients (61%), whereas the remaining fractures were identified only on MRI (Table 1).

**Table 1:** Baseline characteristics of patients with posterior element injury Data shown as mean ± SD

Characteristics	
Age	82 ± 6
Sex	Male 6, Female 12
Location	Thoracolumbar 16, others 2
Bone mineral density (g/cm2)	0.64 ± 0.13
Local kyphosis (degrees)	24 ± 9
Vertebral instability (degrees)	13 ± 6
Spinous process fracture location	One level above 15, Same level 3

Compared with the 171 patients without posterior element injury, patients with posterior element injury had a higher prevalence of thoracolumbar fractures (16/18 (89%) vs. 113/171 (66%), p = 0.048), a larger local kyphotic angle (24 ± 9° vs. 17 ± 8°, p = 0.015), and greater vertebral instability (13 ± 6° vs. 9 ± 5°, p < 0.01).

At three months after BKP, eight patients (44%) had an NRS pain score ≥4, and AVFs occurred in seven patients (39%). Overall, seven patients (39%) met the predefined criteria for a favorable outcome, defined as an NRS score <4 and the absence of AVFs. Comparison of patients with favorable and unfavorable outcomes showed no significant differences in age, sex, history of previous vertebral fracture, fracture location, BMD, or local kyphotic angle (Table 2).

**Table 2:** Comparison of baseline characteristics between patients with favorable and unfavorable outcomes Comparison of baseline characteristics between patients with favorable and unfavorable outcomes. Data are presented as n (%). Categorical variables were compared using Fisher's exact test. A p-value < 0.05 was considered statistically significant. TL - thoracolumbar

Characteristics	Favorable (n=7)	Unfavorable (n=11)	p-value
Age (≧80 years), n (%)	3 (43)	9 (82)	0.09
Sex (female), n (%)	5 (71)	7 (64)	0.73
Past history of vertebral fracture (+), n (%)	4 (57)	5 (46)	0.63
Location (TL), n (%)	6 (86)	9 (82)	0.73
Bone mineral density (<0.7g/cm2), n(%)	3 (43)	7 (64)	0.83
Local kyphosis (≧20 degrees), n (%)	4 (57)	9 (82)	0.26
Vertebral instability (≧10 degrees), n (%)	2 (29)	10 (91)	0.006

Vertebral instability of ≥10° was significantly more common in the unfavorable outcome group than in the favorable outcome group (91% vs. 29%, p = 0.006).

During the postoperative follow-up period (median, 17 months; interquartile range, 12-44 months), no patient underwent revision surgery. There was no significant difference in the local kyphotic angle between the preoperative evaluation and the final follow-up (0 ± 7°, p = 1.00).

Case one - unfavorable outcome

**Figure 2:**
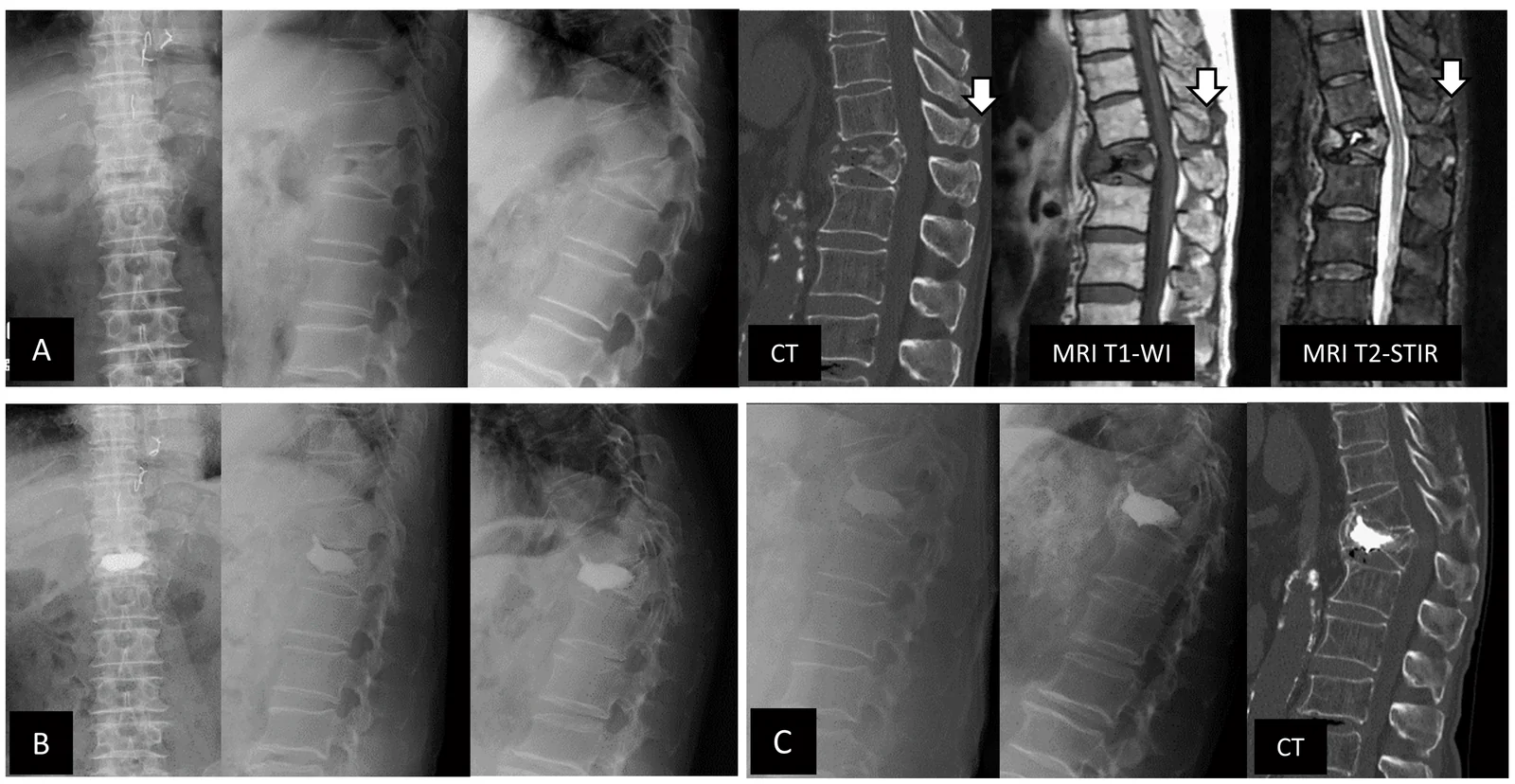
Preoperative and postoperative images of an 80-year-old male with a T12 vertebral fracture A) Preoperative images: from the left - radiographs (anteroposterior, supine lateral, seated lateral), computed tomography (CT), magnetic resonance imaging (MRI) (T1-WI, T2-STIR). White arrows indicate the spinous process fracture line; B) One week postoperative: from the left - radiographs (anteroposterior, supine lateral, seated lateral); C) Two years postoperative: from the left - radiographs (supine lateral, seated lateral), CT (sagittal view).

An 80-year-old male presented with a T12 vertebral fracture five months after the injury. The kyphotic angle was 20° in the supine position and 40° in the sitting position, showing a vertebral instability of 20°. MRI showed signal changes at the spinous process of T11, and CT confirmed a fracture line (Figure 2). BKP was performed with 6 ml of cement. Subsidence was observed in the cranial vertebra, and instability remained between T11 and T12. The patient continued to experience low back pain without evidence of cement mobility. At the two-year follow-up, the vertebrae fused with bridging at the anterior vertebral body. Low back pain was still reported at the final follow-up.

Case two - favorable outcome

**Figure 3:**
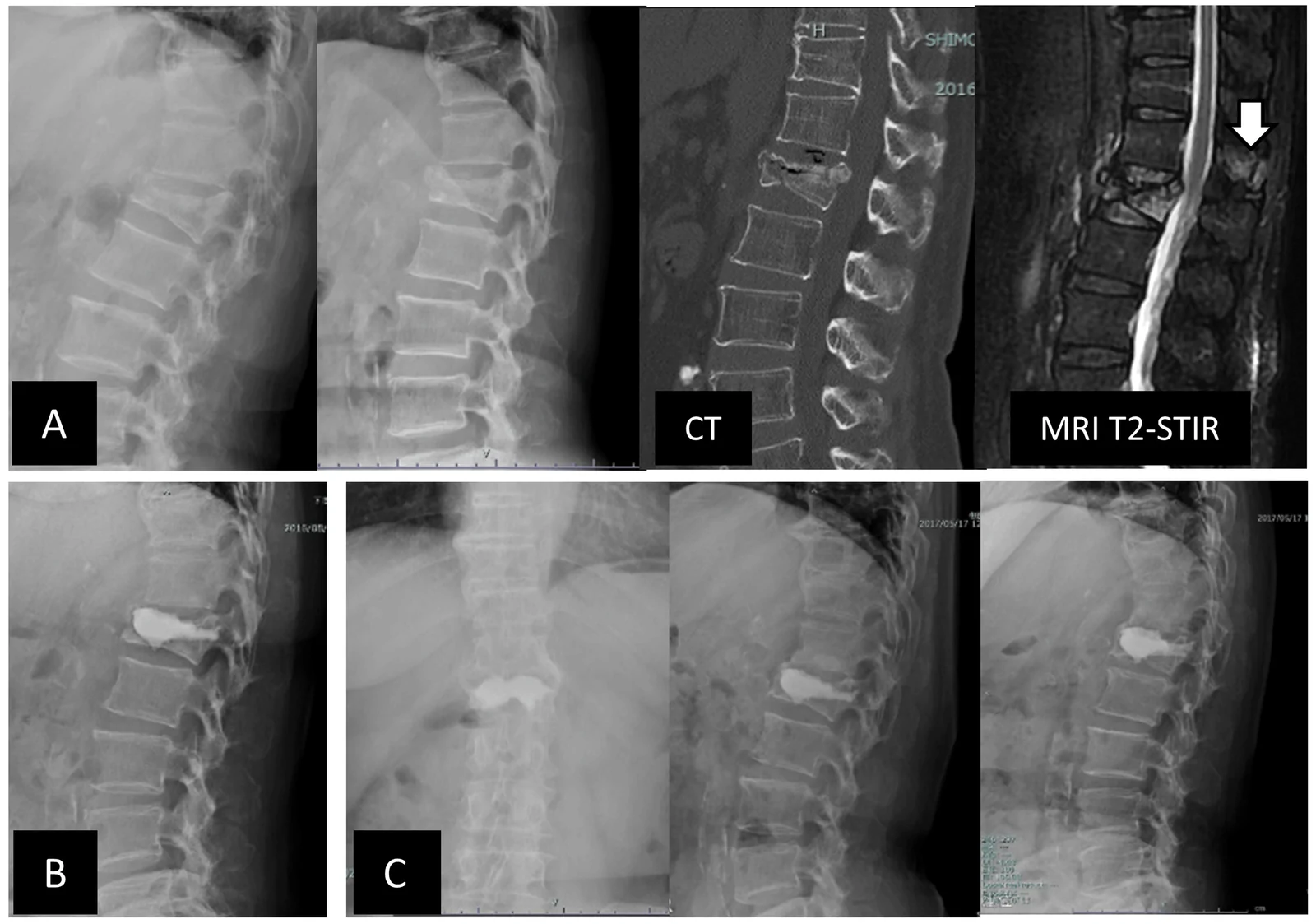
Preoperative and postoperative images of a 74-year-old female with a T12 vertebral fracture A: Preoperative images: from the left - radiographs (supine lateral, seated lateral), computed tomography (CT), magnetic resonance imaging (MRI) (T2-STIR). White arrow indicates the spinous process fracture line; B) One-week postoperative radiograph (lateral view in the lateral decubitus position); C) One year postoperative: from the left - radiographs (anteroposterior, supine lateral, seated lateral).

A 74-year-old female presented with a T12 vertebral fracture one month after the injury. The kyphotic angle in the supine position was 13°, and 19° in the sitting position, showing a vertebral instability of 6°. MRI showed signal changes at the spinous process of T11, but no clear fracture line was visible on CT (Figure 3). BKP was performed with 7.7 ml of cement, and pain was relieved. At the one-year follow-up, the anterior vertebrae fused with bridging. The patient reported no residual back pain at the final follow-up.

## Discussion

We investigated the surgical outcomes of BKP for OVFs with posterior element injury. The results indicated that while BKP can be effective in some cases with posterior element injury, outcomes were poor in cases with significant instability.

Although vertebroplasty and BKP are established treatments for OVFs, there remains debate over the degree of vertebral body compromise or instability for which these interventions are considered appropriate. Although many OVFs initially occur without middle or posterior column injury, delayed nonunion may eventually lead to posterior element injury (Figure 1) [10]. Some reports suggest that BKP can be effective even in cases with middle column injury, but there have been few reports regarding BKP in patients with posterior element injury. It remains unclear whether BKP alone can address such cases. In our study, 56% of patients achieved an NRS score <4, indicating that BKP provided satisfactory pain relief in many patients despite the presence of posterior element injury. These pain outcomes were comparable to those reported in previous studies using vertebroplasty [8], suggesting that posterior element injury does not necessarily compromise the analgesic effect of BKP. However, pain relief and structural outcomes should be interpreted separately. Because AVF is one of the most common complications after BKP and may adversely affect subsequent clinical outcomes, we defined a favorable outcome as both an NRS score <4 and the absence of AVFs. Using this composite outcome, only 39% of patients achieved a favorable result. Thus, pain relief alone may not adequately reflect overall clinical success after BKP. The incidence of AVFs was 39% at three months postoperatively, which notably exceeds the 5-23% incidence reported in previous studies [11-13]. One possible explanation is that local kyphosis caused by the fractured vertebra alters spinal biomechanics and increases stress on adjacent vertebrae [14-15]. In our cohort, these patients exhibited significantly greater local kyphosis and vertebral instability, which may have contributed to the high incidence of AVFs. Furthermore, AVF-related kyphosis may exacerbate pre-existing deformity, as illustrated in case one, suggesting that BKP alone is not ideal for alignment correction in such cases. Therefore, when evaluating the effectiveness of BKP in patients with posterior element injury, both symptomatic improvement and structural outcomes should be considered. In our study cohort, there was no significant difference between pre- and postoperative local kyphotic angles, indicating a limited corrective effect. However, this limitation is not unique to patients with posterior element injuries; several prior studies have reported similarly minimal kyphosis correction following BKP [16]. Since posterior element injury often accompanies marked kyphosis, alternative approaches may be needed if we aim to improve alignment.

It is also worth emphasizing the importance of appropriate case selection. We did not intentionally treat cases with posterior element injury using BKP alone. Rather, during our investigation of postoperative outcomes following BKP, we re-evaluated preoperative MRIs for all cases and found that some patients had unrecognized posterior element injuries. None of the posterior element injuries in our cohort were visible on plain radiographs. Moreover, only 61% of spinous process fractures were detected on CT, despite using 3 mm sagittal slices. This underscores the importance of preoperative MRI evaluation. However, the spinous process fractures lacked obvious surrounding bone marrow edema signal, suggesting that these injuries were not acute but may have developed progressively due to persistent mechanical stress or delayed union over time. These injuries are easily overlooked without careful review. Because occult posterior element injuries are difficult to detect, it is possible that some patients with poor BKP outcomes in previous studies had unrecognized posterior instability, which may partially explain the wide variation in reported rates of AVFs. Notably, posterior element injuries were often associated with marked instability at the thoracolumbar junction, where particular attention is warranted.

This study has several limitations. First, it reports only short-term outcomes at three months postoperatively, so the long-term effects of residual kyphosis and instability remain unclear. Second, functional outcomes such as activities of daily living (ADL) were not assessed; only pain severity via NRS was evaluated. Third, because of the small sample size, the statistical analyses should be considered exploratory, and the findings should not be interpreted as definitive predictors. The relatively small sample size also prevented multivariate analysis, limiting the ability to identify independent predictors of poor outcomes. In addition, this was a single-center study. Because MRI findings were retrospectively assessed, observer bias cannot be completely excluded. Therefore, the generalizability of our findings may be limited. Despite these limitations, our study provides preliminary evidence in an under-investigated clinical subset and may serve as a foundation for larger prospective studies.

## Conclusions

BKP may be an effective treatment option for selected patients with OVFs and posterior element injury, particularly when vertebral instability is mild. However, patients with marked vertebral instability, especially at the thoracolumbar junction, appeared to have poorer outcomes after BKP. These findings suggest that additional stabilization procedures, including fusion surgery, may be considered in such patients, although further prospective studies are required before definitive treatment recommendations can be made. Preoperative MRI may be useful for detecting occult posterior element injuries that could influence treatment planning.
